# Supplementary material for: Structural elements of a pH-sensitive inhibitor binding site in NMDA receptors
Source: Nat Commun. 2019 Jan 18;10:321. doi: 10.1038/s41467-019-08291-1 (PMC6338780; doi:10.1038/s41467-019-08291-1)
Supplement: Supplementary file 1 — Supplementary Information [file 41467_2019_8291_MOESM1_ESM.pdf]

Supplementary Information

Structural Elements of a pH-Sensitive Inhibitor Binding Site in NMDA Receptors.

Michael C. Regan[1], Zongjian Zhu[2,3], Hongjie Yuan[2], Scott J. Myers[2], Dave S. Menaldino[4], Yesim A. Tahirovic[4], Dennis C Liotta[4], Stephen F. Traynelis[2], and Hiro Furukawa[1].

This file contains:

Supplementary Figures 1 to 8

Supplementary Table 1 to 2

a

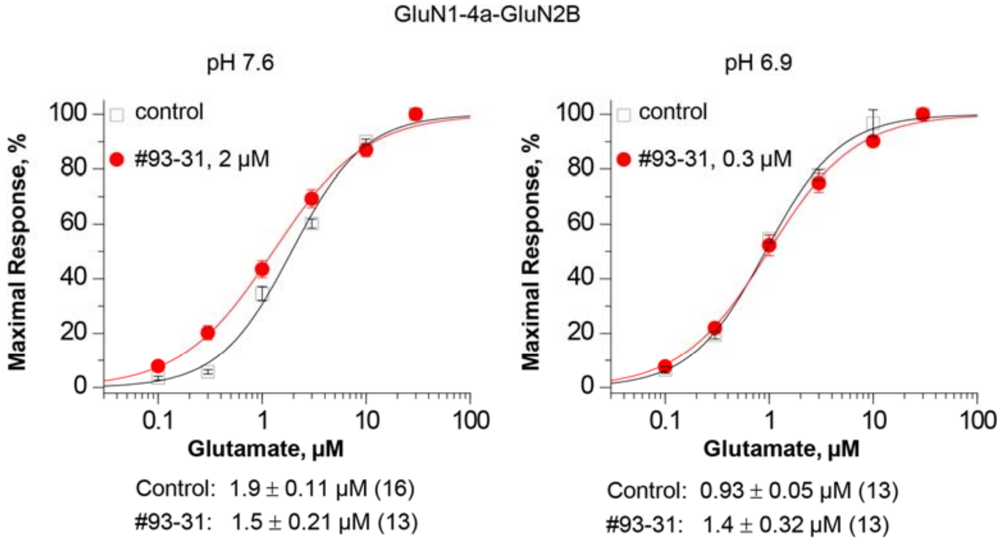

b

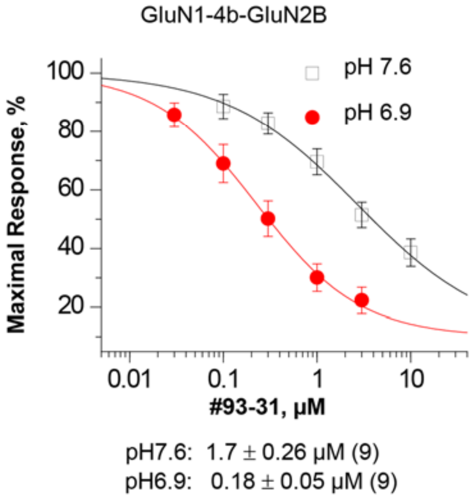

Supplemental Figure 1 | Binding properties of 93-31. a, Glutamate dose-response curves (in the presence of 100 μM glycine) from TEVC experiments in the presence (red filled circles) or absence (empty squares) of 2 μM of 93-31 at pH 7.6 (*left panel*) and 0.3 μM at 6.9 (*right panel*) for activation of wild type GluN1-4a/GluN2B NMDA receptor. **b,** Concentration-response curves for inhibition of wild type GluN1-4b/GluN2B NMDA receptor by 93-31 at pH 7.6 (empty squares) and at pH 6.9 (red circles) (Please also see **Table 2**). The fitted minimums were set as 10%. Symbols and error bars represent mean ± S.E.M.

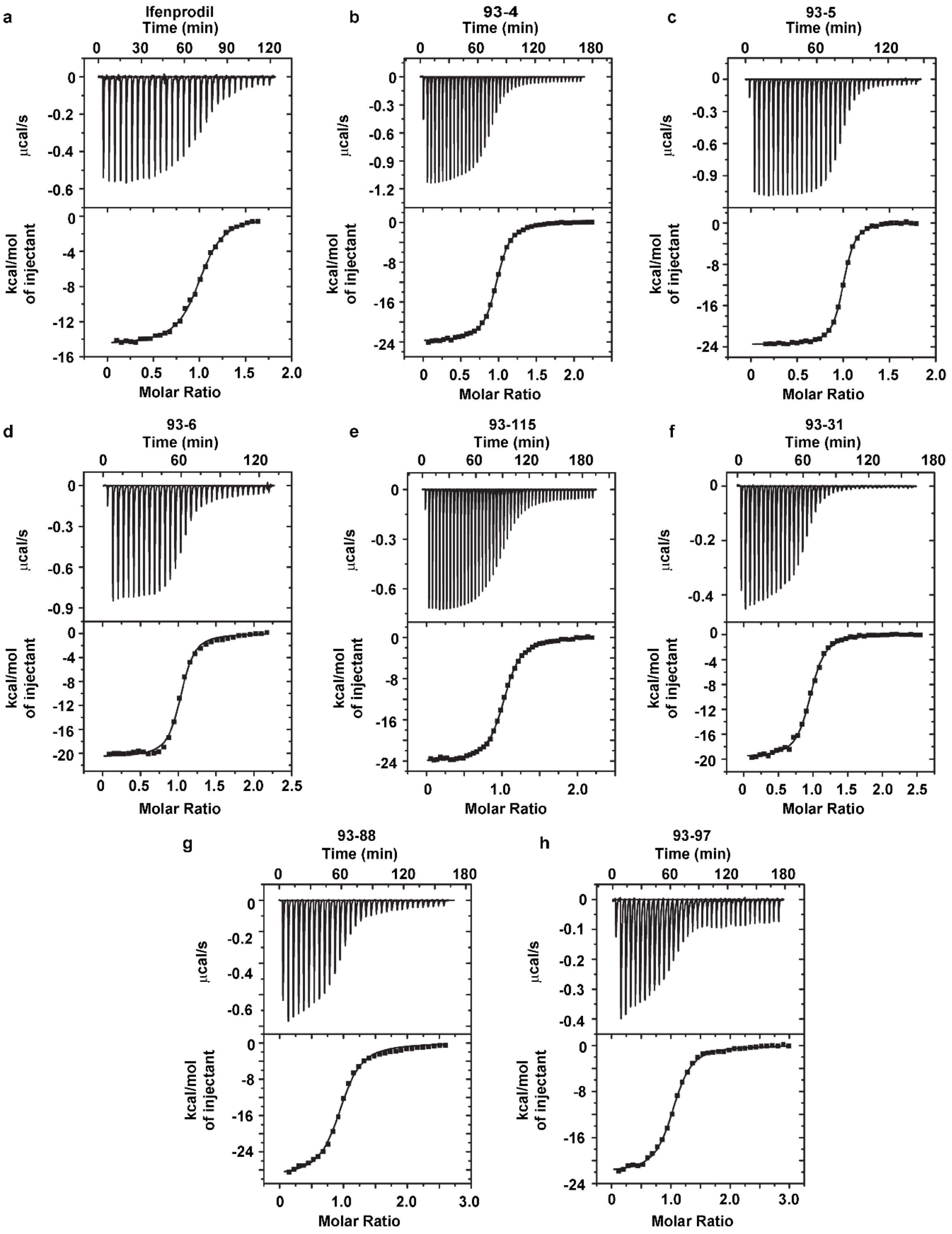

Supplemental Figure 2 | Representative ITC isotherms at pH 7.6. a-h The isolated GluN1a-GluN2B ATD was titrated with ligands as described in **Methods**; the results of triplicate experiments are listed in **Table 1**.

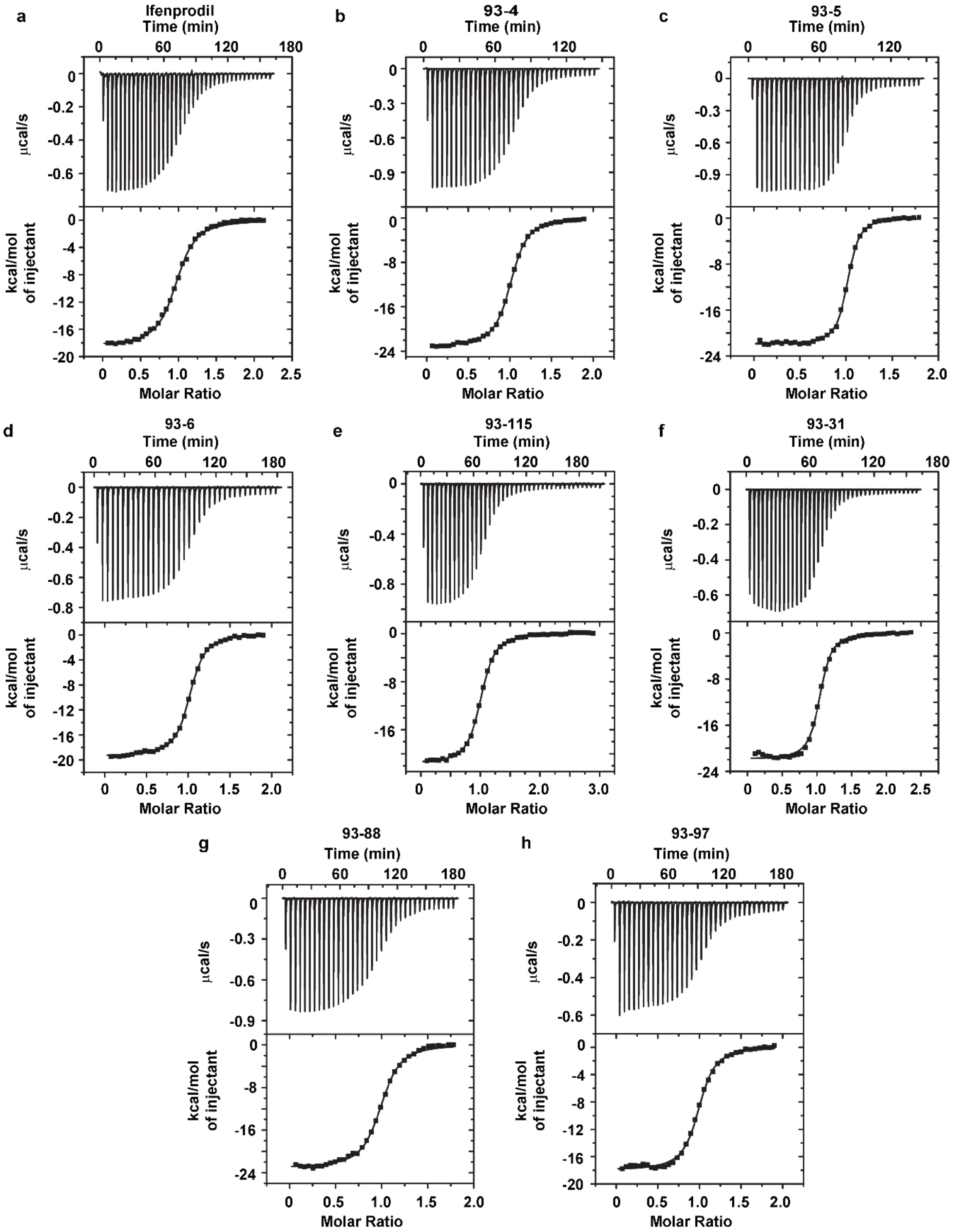

Supplemental Figure 3 | Representative ITC isotherms at pH 6.5. a-h The isolated GluN1a-GluN2B ATD was titrated with the given ligands as described in **Methods**; the results of triplicate experiments are listed in **Table 1**.

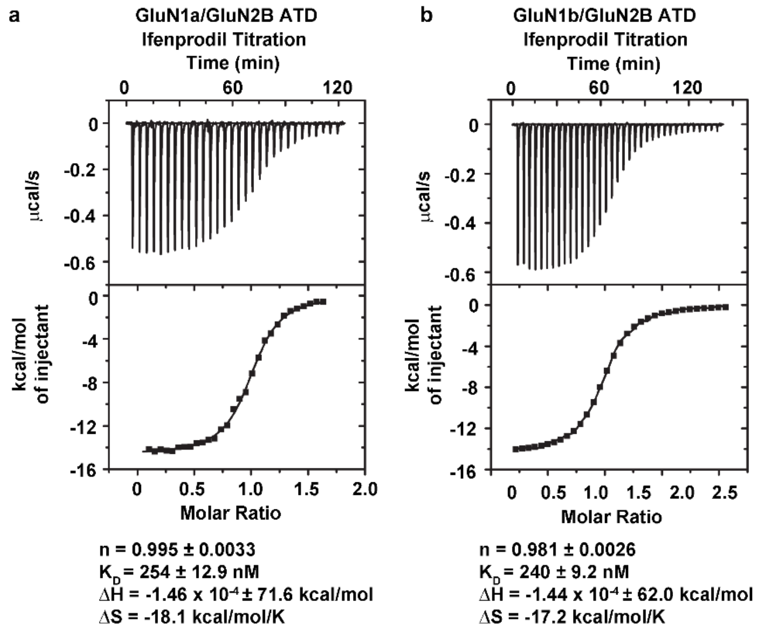

Supplemental Figure 4 | Comparison of ligand binding between the GluN1a-GluN2B ATD from HEK cells and GluN1b-GluN2B ATD from High5 cells. Despite extensive efforts, we were unable to obtain crystals of the GluN1a-GluN2B ATD expressed in HEK cells; however, initial experiments demonstrated no substantial differences in the ATD/ifenprodil binding interaction between the GluN1a-GluN2B ATD expressed in HEK cells (**a**) and the GluN1b-GluN2B ATD expressed in High 5 cells that we used for crystallization studies (**b**).

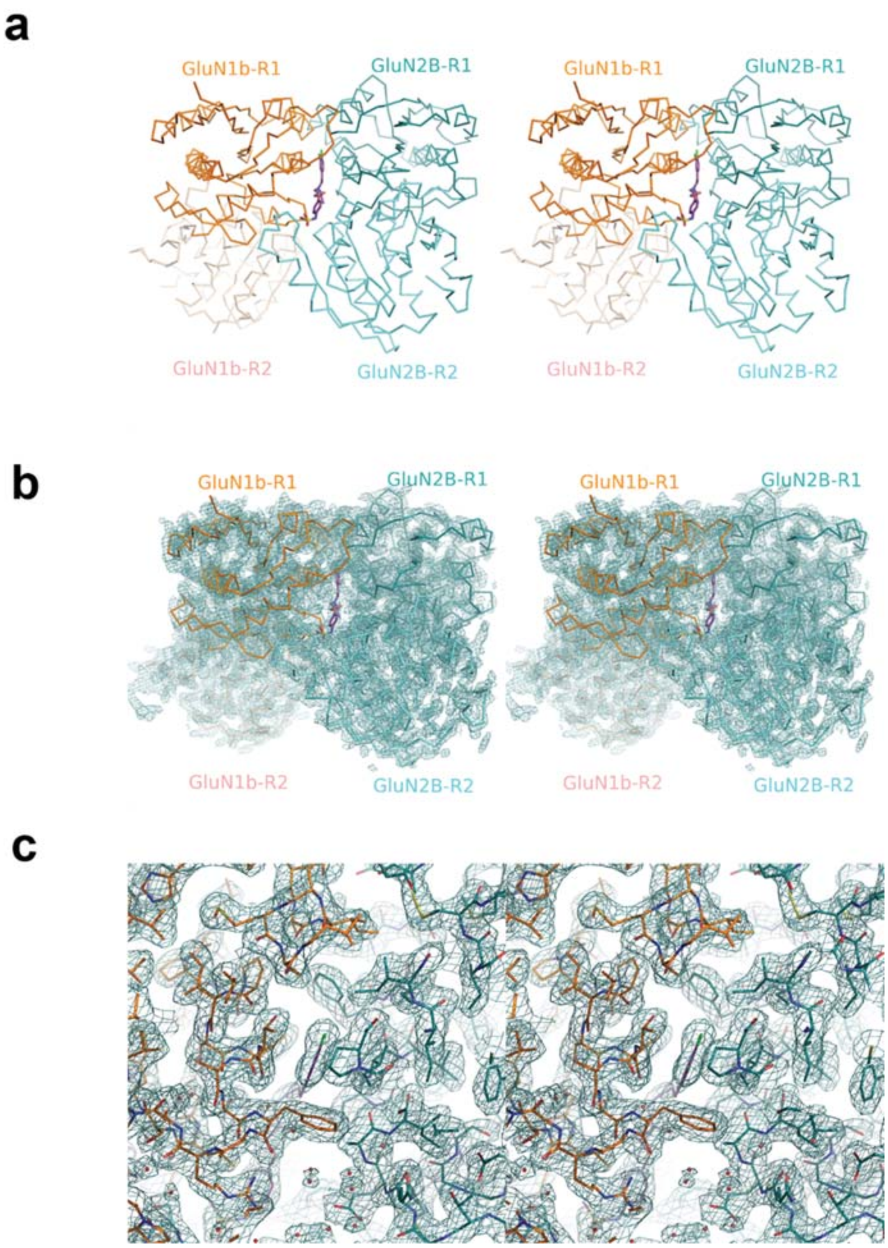

Supplemental Figure 5 | Crystal structure of GluN1b-GluN2B ATD complexed to 93-31. (a) Stereoview of the GluN1b-GluN2B ATD structural model. The purple stick at the GluN1b-GluN2B ATDs represents 93-31. **(b)** The *2Fo-Fc* electron density map (mesh) countered at 1.5σ in the same orientation in *panel* a. **(c)** The *2Fo-Fc* electron density map (mesh) countered at 1.5σ at the GluN1b-GluN2B heterodimer interface looking down from the 'top' of the binding site of 93-31 (purple stick) showing fine structural details such as oxygen atoms from waters (red spheres). Color codes for the structural models are as in Fig. 3.

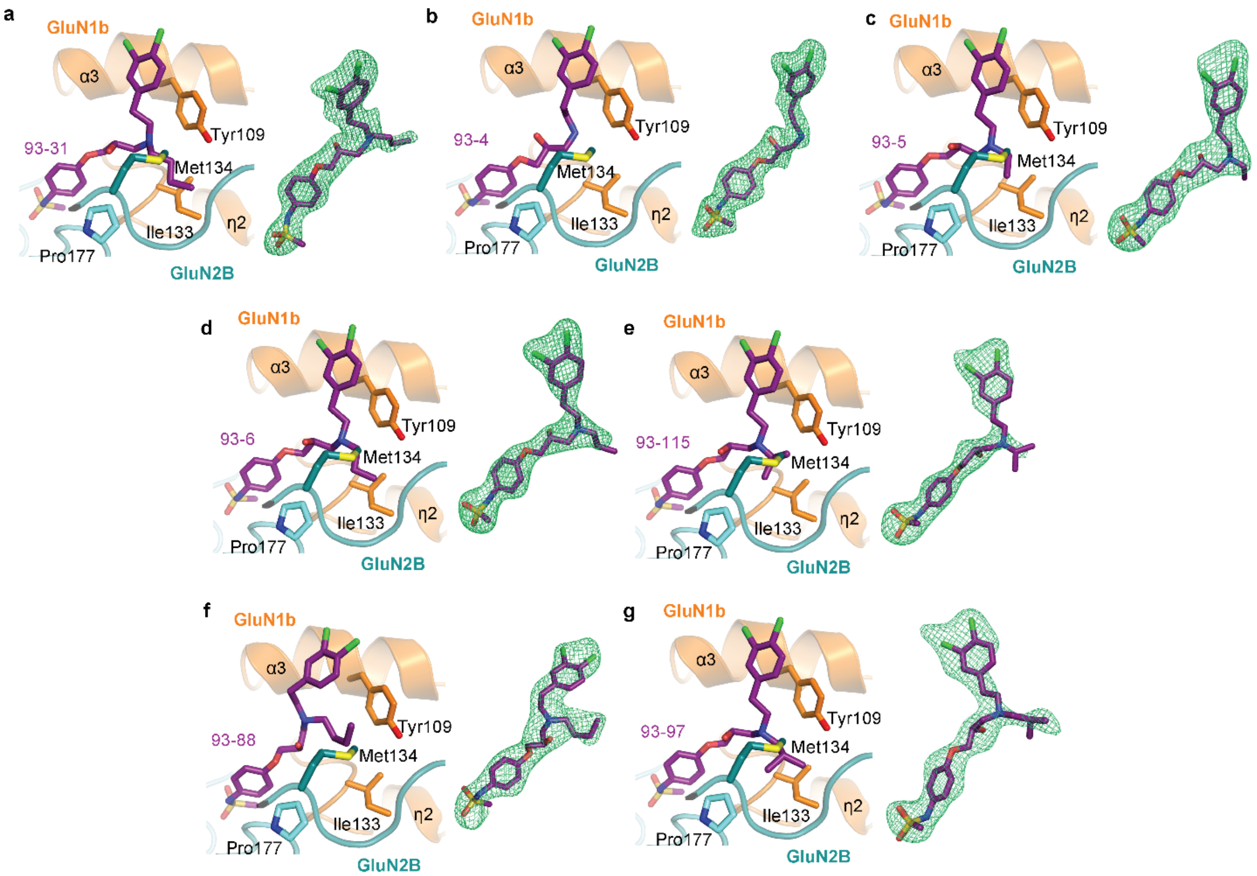

Supplemental Figure 6 | Binding poses and electron densities for 93-series ligands. a-g. The 93-series negative allosteric modulators (purple sticks) bind at the GluN1-GluN2B ATD dimer interface in a similar fashion, with the exception of 93-88 (**f**), which, as the *(R)* enantiomer, adopts a rather different orientation. Electron densities (green mesh) were generated from the F_o-F_c omit map, contoured at 3 σ. For clarity, water molecules at the binding site have been omitted.

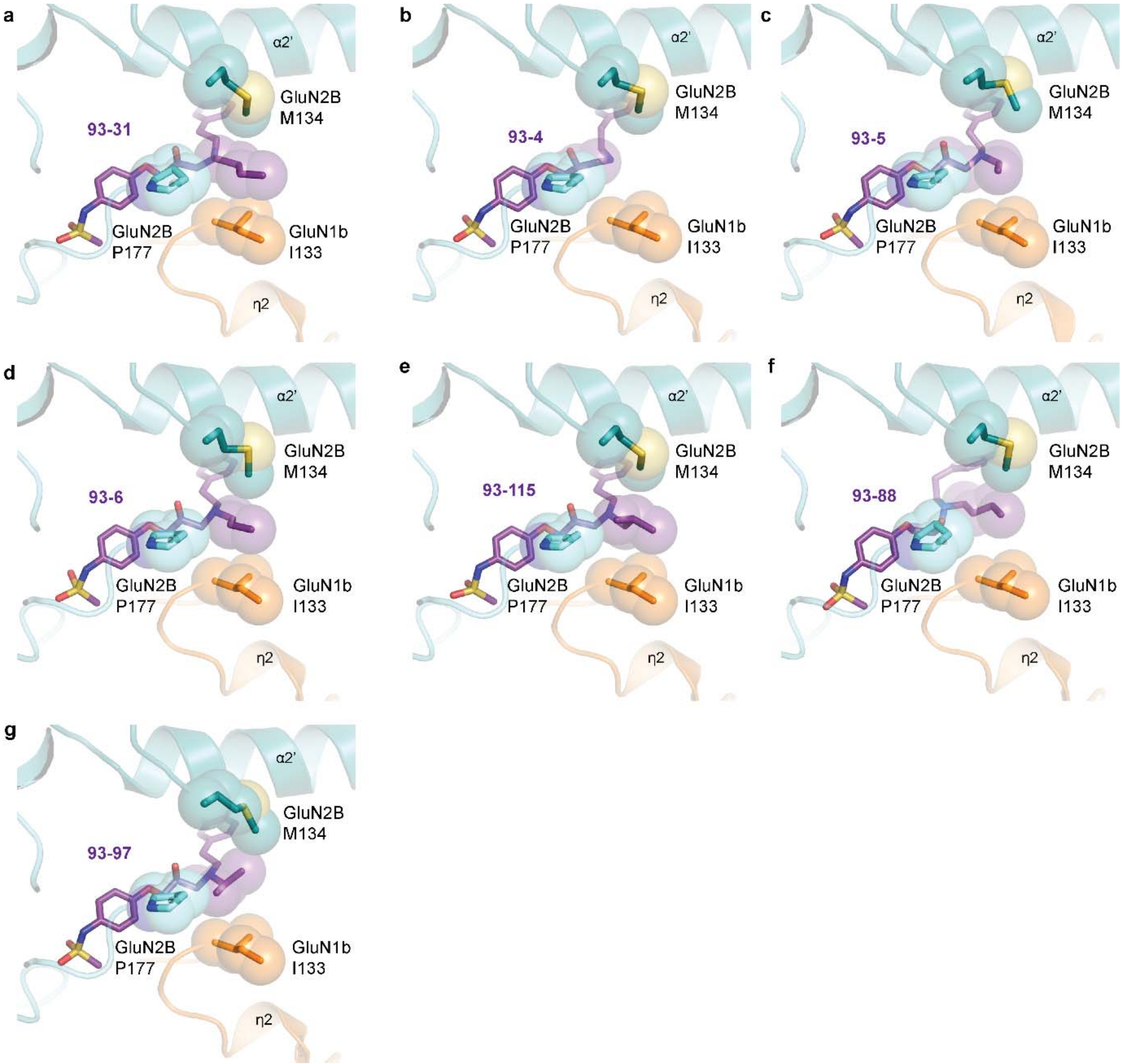

Supplemental Figure 7 | Hydrophobic interactions of the 93-series ligands. a-g. The N-alkyl substitution present in 93-series compounds forms hydrophobic interactions with GluN1b(Ile133), GluN2B(Met134), and GluN2B(Pro177).

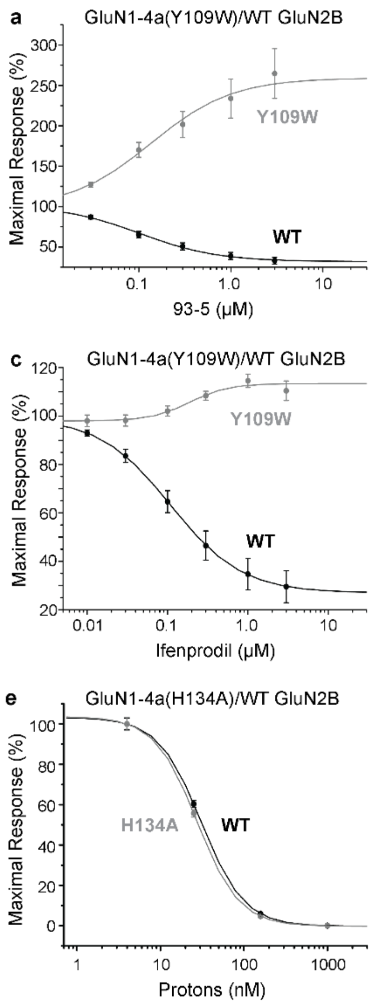

Supplemental Figure 8 | GluN1 potentiating mutants. a-d. TEVC recordings with the GluN1-4a(His134Ala) and GluN1-4a(Tyr109Trp) mutants co-expressed with wild type GluN2B at pH 7.6. Both mutants convert 93-5 to a potentiator of the NMDA receptor, and completely eliminate the inhibitory effect of ifenprodil. **e-f.** Neither mutant has an effect on NMDA receptor proton sensitivity. Error bars represent mean ± S.E.M.

Supplementary Table 1 | Data collection and refinement statistics.

	**ATD + 93-4**	**ATD + 93-5**	**ATD + 93-6**	**ATD + 93-115**	**ATD + 93-31**	**ATD + 93-88**	**ATD + 93-97**
**Data collection**							
Space group	C2	C2	C2	C2	C2	C2	C2
Unit cell dimensions							
a, b, c (Å)	267.95, 59.90, 145.29	268.43, 60.63, 145.39	268.59, 59.84, 146.02	268.13, 59.59, 145.50	268.62, 59.60, 145.91	268.13, 59.61, 145.53	268.42, 59.54, 146.11
α, β, γ (º)	90, 116.69, 90	90, 116.42, 90	90, 116.86, 90	90, 116.62, 90	90, 117.10, 90	90, 116.86, 90	90, 116.69, 90
Resolution (Å)	35.00-2.10 (2.14)	29.88-2.72 (2.79)	50.00-2.31 (2.35)	35.00-2.67 (2.72)	35.00-2.27 (2.32)	35.00-2.60 (2.64)	35.00-2.58 (2.62)
R_{merge}	4.8 (104)	6.5 (95.8)	9.2 (60.6)	8.3 (76.6)	6.8 (109)	9.1 (65.9)	11.4 (86.0)
I/σI	24.9 (1.0)	14.4 (1.4)	13.6 (1.3)	11.5 (1.04)	15.6 (1.0)	8.3 (1.0)	10.6 (1.14)
Completeness (%)	98.8 (96.8)	98.6 (93.9)	91.0 (48.1)	89.7 (89.8)	98.6 (97.3)	88.6 (63.8)	96.2 (89.9)
Redundancy	4.0 (3.2)	3.5 (3.2)	3.6 (1.9)	2.3 (2.0)	3.4 (3.0)	2.0 (1.6)	4.4 (4.0)
**Refinement**							
Resolution (Å)	25.00-2.10	25.00-2.72	25.00-2.31	25.00-2.66	25.00-2.27	25.00-2.60	25.00-2.58
No. reflections	95,244	53,230	67,430	44,140	74,940	44,677	53,795
R_{work}/R_{free}	0.19/0.22	0.19/0.23	0.19/0.23	0.19/0.23	0.20/0.23	0.19/0.23	0.19/0.22
No. atoms							
Protein	11,118	11,149	10,860	11,027	11,129	11,063	11,123
93-series Ligand	54	58	60	60	62	62	62
Na	2	2	2	2	2	2	2
Cl	12	8	11	11	12	11	9
Water	339	178	317	232	333	243	241
B-factors ($Å^2$)							
Protein	40.61	78.30	44.81	52.62	41.85	54.68	51.69
93-series Ligand	38.02	58.83	31.36	43.98	39.81	64.90	51.67
Na	21.98	54.65	29.31	33.46	24.08	33.79	35.60
Cl	60.48	94.19	59.94	69.89	61.05	70.62	67.18
Water	32.12	56.59	33.98	37.42	33.84	39.45	37.81
R.M.S. deviations							
Bond lengths (Å)	0.006	0.002	0.001	0.001	0.004	0.001	0.001
Bond angles (º)	0.372	0.380	0.363	0.362	0.800	0.346	0.389

All datasets were collected from a single crystal. Values in parentheses are for the highest-resolution shell.

Supplemental Table 2 | Mutagenesis primers used in this study for TEVC experiments

Mutation	Forward Primer Sequence	Reverse Primer Sequence
GluN1-4a (S108A)	CACTCCCACCCCTGTCGCCTACACAGCTGGCTTC	GAAGCCAGCTGTGTAGGCGACAGGGGTGGGAGTG
GluN1-4a (Y109A)	CCCACCCCTGTCTCCGCCACAGCTGGCTTCTAC	GTAGAAGCCAGCTGTGGCGGAGACAGGGGTGGG
GluN1-4a (Y109W)	CCCACCCCTGTCTCCTGGACAGCTGGCTTCTAC	GTAGAAGCCAGCTGTCCAGGAGACAGGGGTGGG
GluN1-4a (I133A)	CTACTCTGACAAGAGTGCCCACCTGAGTTTCCTTCG	CGAAGGAAACTCAGGTGGGCACTCTTGTCAGAGTAG
GluN1-4a (H134A)	CTACTCTGACAAGAGTATCGCCCTGAGTTTCCTTCGCACG	CGTGCGAAGGAAACTCAGGGCGATACTCTTGTCAGAGTAG
GluN2B(M134A)	GGCTCATCTATGATAGCGGCGGATAAGGATGAG	CTCATCCTTATCCGCCGCTATCATAGATGAGCC
GluN2B(D136A)	CATCTATGATAATGGCGGCTAAGGATGAGTCCTCCATG	CATGGAGGACTCATCCTTAGCCGCCATTATCATAGATG
GluN2B(P177A)	CGTCACCACCTACTTCGCTGGCTACCAGGAC	GTCCTGGTAGCCAGCGAAGTAGGTGGTGACG
GluN2B(P177G)	CGTCACCACCTACTTCGGTGGCTACCAGGAC	GTCCTGGTAGCCACCGAAGTAGGTGGTGACG
GluN2B(E236A)	CCTTTATTGCACGAAGGAGGCAGCCACCTACATTTTTG	CAAAAATGTAGGTGGCTGCCTCCTTCGTGCAATAAAGG
GluN2B(E236Q)	CCTTTATTGCACGAAGGAGCAAGCCACCTACATTTTTG	CAAAAATGTAGGTGGCTTGCTCCTTCGTGCAATAAAGG
